# Diagnostic disparity and identification of two *TNNI3* gene mutations, one novel and one arising de novo, in South African patients with restrictive cardiomyopathy and focal ventricular hypertrophy

**DOI:** 10.5830/CVJA-2015-019

**Published:** 2015

**Authors:** Jomien M Mouton, Craig J Kinnear, Johanna C Moolman-Smook, Philip G Herbst, Adriano S Pellizzon, Althea Goosen, Paul A Brink

**Affiliations:** SA MRC Centre for Tuberculosis Research, DST /NRF Centre of Excellence for Biomedical Tuberculosis Research, Division of Molecular Biology and Human Genetics, Faculty of Medicine and Health Sciences, Stellenbosch University, Cape Town, South Africa; SA MRC Centre for Tuberculosis Research, DST /NRF Centre of Excellence for Biomedical Tuberculosis Research, Division of Molecular Biology and Human Genetics, Faculty of Medicine and Health Sciences, Stellenbosch University, Cape Town, South Africa; SA MRC Centre for Tuberculosis Research, DST /NRF Centre of Excellence for Biomedical Tuberculosis Research, Division of Molecular Biology and Human Genetics, Faculty of Medicine and Health Sciences, Stellenbosch University, Cape Town, South Africa; Division of Cardiology, Department of Medicine, Faculty of Medicine and Health Sciences, Tygerberg Academic Hospital, Stellenbosch University, Cape Town, South Africa; Department of Medicine, Faculty of Medicine and Health Sciences, Stellenbosch University, Cape Town South Africa; Department of Medicine, Faculty of Medicine and Health Sciences, Stellenbosch University, Cape Town South Africa; Department of Medicine, Faculty of Medicine and Health Sciences, Stellenbosch University, Cape Town South Africa

**Keywords:** hypertrophic cardiomyopathy, restrictive cardiomyopathy, troponin I, echocardiography, disease-causing mutation

## Abstract

**Introduction:**

The minimum criterion for the diagnosis of hypertrophic cardiomyopathy (HCM) is thickening of the left ventricular wall, typically in an asymmetrical or focal fashion, and it requires no functional deficit. Using this criterion, we identified a family with four affected individuals and a single unrelated individual essentially with restrictive cardiomyopathy (RCM). Mutations in genes coding for the thin filaments of cardiac muscle have been described in RCM and HCM with ‘restrictive features’. One such gene encodes for cardiac troponin I (*TNNI3*), a sub-unit of the troponin complex involved in the regulation of striated muscle contraction. We hypothesised that mutations in *TNNI3* could underlie this particular phenotype, and we therefore screened *TNNI3* for mutations in 115 HCM probands.

**Methods:**

Clinical investigation involved examination, echocardiography, chest X-ray and an electrocardiogram of both the index cases and close relatives. The study cohort consisted of 113 South African HCM probands, with and without known founder HCM mutations, and 100 ethnically matched control individuals. Mutation screening of *TNNI3* for disease-causing mutations were performed using high-resolution melt (HRM) analysis.

**Results:**

HRM analyses identified three previously described HCM-causing mutations (p.Pro82Ser, p.Arg162Gln, p.Arg170Gln) and a novel exonic variant (p.Leu144His). A previous study involving the same amino acid identified a p.Leu144Gln mutation in a patient presenting with RCM, with clinical features of HCM. We observed the novel p.Leu144His mutation in three siblings with clinical RCM and varying degrees of ventricular hypertrophy. The isolated index case with the *de novo* p.Arg170Gln mutation presented with a similar phenotype. Both mutations were absent in a healthy control group.

**Conclusion:**

We have identified a novel disease-causing p.Leu144His mutation and a de novo p.Arg170Gln mutation associated with RCM and focal ventricular hypertrophy, often below the typical diagnostic threshold for HCM. Our study provides information regarding *TNNI3* mutations underlying RCM in contrast to other causes of a similar presentation, such as constrictive pericarditis or infiltration of cardiac muscle, all with marked right-sided cardiac manifestations. This study therefore highlights the need for extensive mutation screening of genes encoding for sarcomeric proteins, such as *TNNI3* to identify the underlying cause of this particular phenotype.

## Abstract

Identifying disease and making sophisticated diagnoses at a specialist level is dependent on opportunity. Effective and accurate diagnosis starts with persons presenting to medical concern, their subsequent funneling, levels of awareness and expertise encountered, and available technology. In this article we describe a young woman (index case) with classic features of restrictive cardiomyopathy (RCM) who was referred to us with hypertrophic cardiomyopathy (HCM). Cascade screening identified the same disease in four relatives, in whom the diagnosis for some has changed from tuberculosis (TB) as a cause for pleural effusions, to cor pulmonale, constrictive pericarditis (CP) and RCM as the cause of sarcoidosis or amyloidosis. At the same time, an unrelated young boy presented with a disease profile similar to our index case, but without a history of disease in any other first-degree relative.

In view of the associated focal hypertrophy, we speculated that it could be caused by a mutation in troponin I [I type 3 (*TNNI3*; Genbank accession no. X90780.1)], which has been implicated in cases of unexplained RCM and/or HCM with ‘restrictive features’.^1^-^3^ Mutations in this gene have been described to cause RCM, HCM and dilated cardiomyopathy and specific mutations have on occasion been associated with more than one of these phenotypes.^4^ We focused on screening *TNNI3* in a South African panel of HCM-affected probands for mutations, which included these two probands, using a high-resolution melt (HRM) approach.

## Methods

Institutional approval was granted for this study by the Ethical Review Committee of the Faculty of Medicine and Health Science at Stellenbosch University (N04/C3/062). Informed, written consent was obtained from all participants or, on the behalf of minors/children enrolled in the study, from their next of kin, caretakers or guardians. Clinical investigations were conducted according to the principles expressed in the Declaration of Helsinki.

The study cohort included the two index individuals with RCM with focal ventricular hypertrophy and restrictive features as well as 113 South African HCM-unrelated probands, all previously diagnosed with HCM using standard criteria, with or without known HCM-causing mutations. The two index individuals and first-degree family members underwent a physical examination, 12-lead electrocardiography and transthoracic two-dimensional echocardiography (Doppler, tissue Doppler). Past medical records were attained for deceased family members and, if relevant, in living individuals. Rhythm and conduction abnormalities were defined using established criteria. Echocardiograms were analysed using measurement conventions as outlined in the American Society of Echocardiography^5^ and the British Society of Echocardiography (http://www.bsecho.org/hypertropic-cardiomyopathy/) guidelines for the assessment of patients with HCM.

DNA extraction and mutation analysis: DNA was extracted from peripheral blood obtained from the participants as previously described.^6^ A control group consisted of anonymous blood samples from 100 mixed-ancestry individuals of varying age and gender obtained from the Western Province Blood Transfusion Services.

PCR amplification: the TNNI3 gene reference sequence (accession number: NM_000363.4) was obtained from NCBI Entrez Nucleotides Database (http://www.ncbi.nlm.nih.gov/nucleotide/). All primers were designed using Integrated DNA Technologies Software, Primer Quest (http://www.idtdna.com). Primer sequences are given in Table 1. The NCBI basic local alignment search tool (BLAST) (http://www.ncbi.nlm.nih.gov/BLAST/) was used to examine primer specificity.

**Table 1:** Oligonucleotide primers used for the amplification of relevant exons in the *TNNI3* gene

*Position (exon)*	*Primers (5′-3′)*	*T_m_ (C°)*	*T_a_ (C°)*	*Size (bp)*
1F	CCGTTATCTGGCATAGTGG	56.6	54	338
1R	AGAGTCCCTACGCCTACCT	55.5		
2_3F	GACACAGCCCACCACTAA	55.3	54	366
2_3R	ACTCCCAGGGTCTTGGAT	56.8		
4F	ACTCAGGGCTCAAGTTGG	56.2	54	239
4R	CACCCATTCTCAAGCTCC	56.6		
5F	CACGCCTGGTCTTTATCC	56.6	54	222
5R	AGAAACCTCGCATCCTTG	56.3		
6F	CCCAACAACACACACCAC	56.4	54	177
6R	AAGTCCCAGCCATCTCAC	55.9		
7F	GGAAATGGAAGGAGAAGTACC	56.7	52	257
7R	CCTCAGCATCCTCTTTCC	55.7		
8F	GGAGACCAAGAAGAGACCC	56.1	54	230
8R	GCCTAAGCCCTGGGTAAT	56.7		

bp: base pairs; C°: degrees Celsius; F: forward, R: reverse; T_a_: annealing temperature; T_m_: melting temperature.

Polymerase chain reaction (PCR) was performed in a reaction mixture consisting of 30 ng of genomic DNA, kapa readymix (Kapa Biosystems Inc, Massachusetts, USA) (1X Kapa buffer, 1.5 mM MgCl_2_, 200 μM dNTPs, 1.0 U of kapa polymerase enzyme), 0.2 μM of each forward and reverse primer, 5% formamide, 2 μM SYTO9 fluorescent dye (Invitrogen, California, USA), and H_2_O to a final volume of 50 μl. PCR amplifications were performed using the GeneAmp® PCR System 2700 (Applied Biosystems Inc, California, USA). The standard PCR cycle consisted of initial denaturation at 95°C for three minutes, followed by 35 cycles of denaturation at 95°C for 30 seconds, annealing at the T_a_ for the specific PCR primer sets for 30 seconds, elongation at 72°C for 30 seconds, and a final elongation step at 72°C for five minutes.

HRM analysis: PCR products were subsequently subjected to HRM analysis on a Rotor-Gene 6000 analyser (Corbet Life Sciences, Brisbane, Australia). The samples were held at 50°C for one minute to ensure that the DNA was double stranded before being melted by increasing the temperature from 75 to 95°C in 0.1°C increments. As the DNA separated into single strands, the shift in fluorescence was measured. A characteristic denaturing profile, which is based on the length and GC content of the amplicon, was visualised for each DNA sample.

Nucleotide sequencing: representative DNA samples for each distinct melting curve, identified by HRM analysis, were bi-directionally sequenced using the BigDye® Terminator v3.1 cycle sequencing kit (Applied Biosystems Inc), followed by electrophoresis on an ABI 3130XL genetic analyser (Applied Biosystems Inc). Sequences were analysed using BioEdit sequence alignment editor software v7.0.9.0.^7^ The sequences were aligned to the *TNNI3* reference sequence (accession number: NM_000363.4) in NCBI (http://www.ncbi.nlm.nih.gov/nucleotide/), using the ClustalW v1.4 programme and analysed for mismatches to the reference sequence.

The effect of sequence variants on restriction enzyme recognition sites was determined using restrictionmapper.org, http://insilico.ehu.es/restriction, and NEBcutter, http://tools.neb.com/NEBcutter2/. Restriction enzyme analysis was then used to confirm genotypes, where possible. Digestion reactions consisted of 10 μl of the PCR product, 1 × appropriate restriction enzyme buffer, five units of the restriction enzyme and H_2_O to a final volume of 20 μl. Samples were then incubated at the temperature and for the duration recommended by the manufacturer (New England Biolabs Inc, Massachusetts).

Haplotype construction: haplotypes were typed on different loci with microsatellite markers provided by the Ensemble database. Linkage to a specific region on a certain chromosome was determined with four microsatellite markers, namely (D19S926, D19S418, D19S880, D19S605) for *TNNI3*. The forward primer for each marker was fluorescently labelled with either FAM, HEX or Cy5 for easy genotyping. Each marker was amplified by polymerase chain reaction (PCR) using kapa readymix (Kapa Biosystems Inc) using the GeneAmp® PCR system 2700 (Applied Biosystems Inc) and products were analysed on the ABI sequencer (Applied Biosystems Inc). Genotyping data were scored using the ABI GeneMapper 3.0 software (Applied Biosystems Inc).

## Results

## Clinical features of the proband in pedigree 1 (individual 1.III.3)

**Fig. 1.**
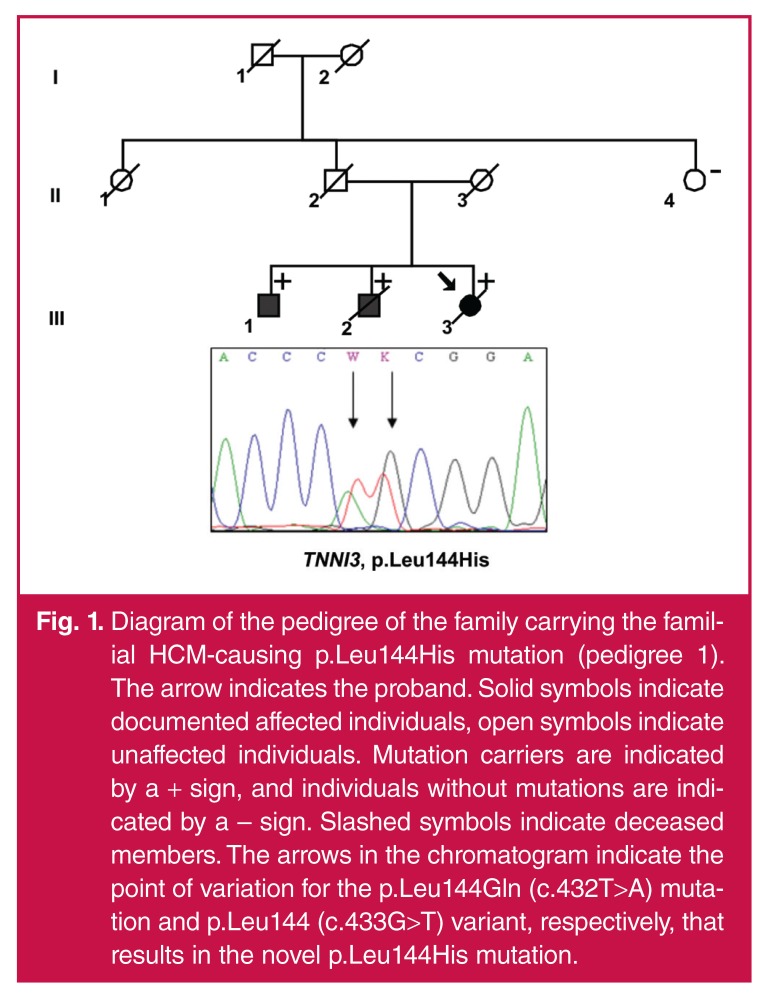
Diagram of the pedigree of the family carrying the familial HCM-causing p.Leu144His mutation (pedigree 1). The arrow indicates the proband. Solid symbols indicate documented affected individuals, open symbols indicate unaffected individuals. Mutation carriers are indicated by a + sign, and individuals without mutations are indicated by a – sign. Slashed symbols indicate deceased members. The arrows in the chromatogram indicate the point of variation for the p.Leu144Gln (c.432T>A) mutation and p.Leu144 (c.433G>T) variant, respectively, that results in the novel p.Leu144His mutation.

A diagnosis of the same disease process was made in a proband and three first-degree family members, namely the father and two brothers of the proband, of whom one was asymptomatic (Fig. 1). While the proband and her two brothers had echocardiograms typically associated with RCM, such as significant bi-atrial dilatation (Table 2) in the presence of non-dilated ventricles with preserved left ventricular systolic function, their presentations differed. The proband, aged 27, had heart failure as evidenced by dyspnoea, a raised jugular venous pressure, marked peripheral oedema and gross hepatomegaly, but with a heart of normal size clinically and on chest X-ray. The latter also showed pulmonary congestion. An apical murmur consistent with mitral regurgitation was present.

**Table 2:** Echocardiographic features of the four affected individuals (*), and the four close family members who screened negative, as comparison

*Measurement*	*1.III.3	*1.III.1	*1.III.2	*2.III.3	*2.III.2*	*2.III.1*	*2.II.8*	*2.II.7*
Rhythm	Atrial flutter	SR	Atrial fibrillation	SR	SR	SR	SR	SR
LA area (cm2)	31	31	32	34	15	15	18	18
RA area (cm2)	29	22	37	46	11	15	11	14
LVED (mm)	41	48	46	40	53	54	55	51
LVEF (%)	54	56	59	31	63	50	62	60
S′ lat (cm/s)	3.6	9	8	4	11	11	11	7
RVED 4C (mm)	33	27	31	30	32	36	29	38
TAPSE (mm)	7	17	10	6	20	20	28	25
Max WT (mm)	14	13	10	21	7	7	8	8
Max WT (position)	LV apex	Mid-LV septum	RV free wall	Mid-LV septum	–	–	–	–
Max RVH (mm)	13	nil	10	9	nil	nil	nil	nil
IVCm/ IVCs (mm/mm)	31/31	16/9	27/26	25/19	x	x	x	x
E (cm/s)	44	68	74	48	121	88	89	69
A (cm/s)	–	25	–	21	44	68	96	54
E/A	–	2.7	–	2.3	2.8	1.3	0.9	1.3
E–DT (ms)	96	111	135	105	209	210	217	207
e′ lat (cm/s)	4	7	7.6	3.5	26	18	17	14
e′ sep (cm/s)	3	x	x	2	x	x	x	x
E/e′ lat	11	9.7	9.7	13.7	4.6	4.9	5.3	4.9

A: maximal transmitral A-wave velocity measured with pulsed-wave Doppler; E: maximal transmitral E-wave velocity with pulsed-wave Doppler; E/A: ratio of E to A; E–DT: transmitral E-wave deceleration time in ms; E/e′ lat: ratio of E wave to e′ lat; e′ lat: early diastolic pulsed-wave tissue Doppler velocity measured at the lateral mitral valve annulus; e′ sep: late diastolic (atrial contraction) pulsed-wave tissue Doppler velocity measured at the septal mitral annulus; LA area: left atrial area; LV: left ventricular area; LVED: left ventricular end-diastolic dimension; LVEF: left ventricular ejection fraction; IVCm/IVCs: the two numbers represent the maximal inferior vena cava diameter and the minimum inferior vena cava diameter, respectively, after a sniff manoeuvre; Max RVH: where right ventricular hypertrophy is present, this denotes the maximal right ventricular wall thickness measurement; Max WT: maximal wall thickness measured in mm; Max WT position: describes the position of measurement of maximal wall thickness; RA area: right atrial area; RV: right ventricular area; RVED 4C: basal right ventricular inflow dimension at end-diastole as measured in the four-chamber view; S′ lat: pulsed-wave tissue Doppler-derived lateral mitral annular systolic velocity; SR: sinus rhythm; TAPSE: tricuspid annular-plane systolic excursion measured by m-mode; x: data not available; –: measurement not applicable.

Despite management involving diuretics, other heart-failure medication and anticoagulation, the disease progressed. Atrial fibrillation ensued and heart failure worsened. Three years after presentation the proband suffered an embolic ilio-femoral occlusion and died. Post-mortem examination showed pulmonary emboli.

## Pedigree 1 clinical analysis

The younger brother of the proband, individual 1.III.2 (Fig. 1) had recurrent admissions over a seven-year period from the age of 25 years, with recurrent isolated pleural effusions, both right and left. Despite being transudates, the effusions were on more than one occasion empirically treated as tuberculous. He was also labelled ‘cor pulmonale’ but no lung disease that could explain such a diagnosis was present. He has since died at the age of 32 with severe right-sided congestion while being evaluated for a heart transplant. The elder brother of the proband, individual 1.III.1 (Fig. 1), was asymptomatic at the time of most recent contact, with an unremarkable clinical examination.

Their father, individual 1.II.2 (Fig. 1)) presented at a similar age as the proband, aged 25 years (in 1978). With the clinical emphasis on right-sided heart failure, he had a sequential series of diagnoses, namely pulmonary hypertension with cardiac failure, CP due to TB, since TB and its various manifestations are extremely common in South Africa,^8^ and finally RCM (sarcoidosis/amyloidosis). A first cardiac catheterisation was believed to be compatible with CP, but a note drew attention to the absence of an apparent thickened pericardium.

His course was complicated by the onset of atrial fibrillation and he died, aged 28 years, as a consequence of recurrent embolic phenomena with cerebrovascular accidents on more than one occasion, despite anticoagulation. Material from a pericardial, endocardial or tongue biopsy as a possible source of DNA for a molecular diagnosis could not be traced.

## Clinical features of the proband in pedigree 2 (Individual 2.II.3)

**Fig. 3.**
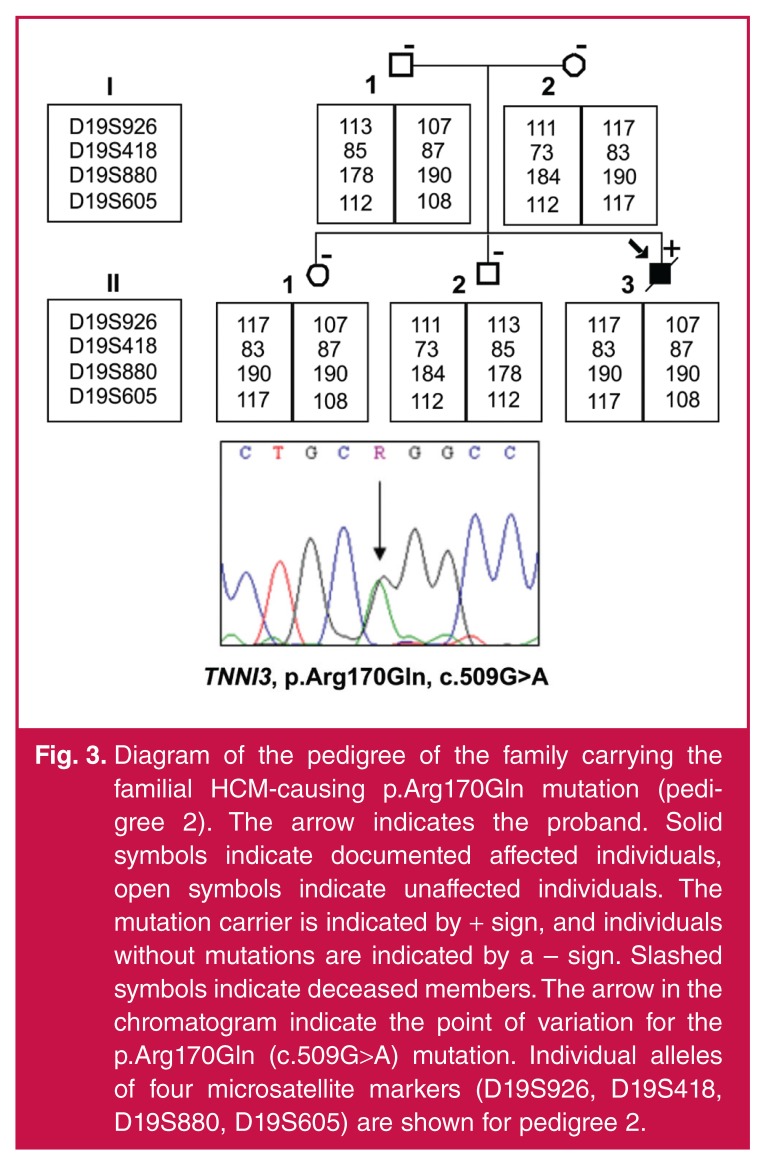
Diagram of the pedigree of the family carrying the familial HCM-causing p.Arg170Gln mutation (pedigree 2). The arrow indicates the proband. Solid symbols indicate documented affected individuals, open symbols indicate unaffected individuals. The mutation carrier is indicated by + sign, and individuals without mutations are indicated by a – sign. Slashed symbols indicate deceased members. The arrow in the chromatogram indicate the point of variation for the p.Arg170Gln (c.509G>A) mutation. Individual alleles of four microsatellite markers (D19S926, D19S418, D19S880, D19S605) are shown for pedigree 2.

Individual 2.II.3 (Fig. 3) presented with signs and symptoms of biventricular cardiac failure at age 15 years. Although clubbed feet were surgically corrected soon after birth, no cardiac anomalies were documented at that time. An electrocardiogram showed sinus rhythm with peaked P waves and a chest X-ray demonstrated a widened cardiothoracic ratio with evidence of pulmonary vascular congestion. His echocardiogram showed markedly dilated atria and non-dilated, small ventricles (Table 2). He was treated for heart failure on admission and was discharged on anti-failure and anti-coagulation medication.

## Pedigree 2 clinical analysis

The nuclear family, comprising the parents, an older sister and older brother (Fig. 3), were normal on physical examination, electrocardiography and echocardiography (Table 2).

## In-depth echocardiographic analysis

**Fig. 2.**
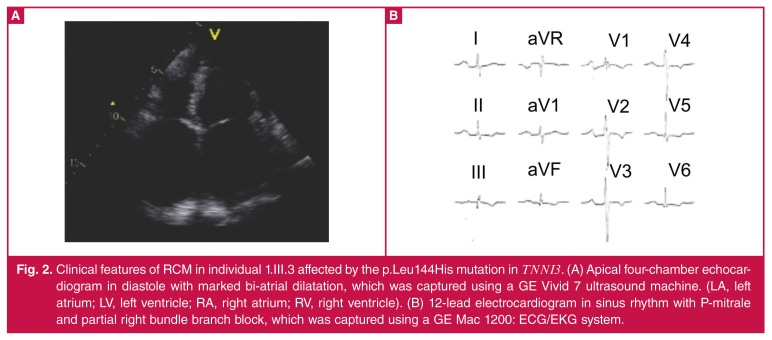
Clinical features of RCM in individual 1.III.3 affected by the p.Leu144His mutation in *TNNI3*. (A) Apical four-chamber echocardiogram in diastole with marked bi-atrial dilatation, which was captured using a GE Vivid 7 ultrasound machine. (LA, left atrium; LV, left ventricle; RA, right atrium; RV, right ventricle). (B) 12-lead electrocardiogram in sinus rhythm with P-mitrale and partial right bundle branch block, which was captured using a GE Mac 1200: ECG/EKG system.

Echocardiographic findings are summarised in Table 2. In pedigree 1, all the siblings had extremely enlarged atriae with relatively small end-diastolic left ventricles with good ejection fractions (Fig. 2). However, in the proband (1.III.3, Fig. 1), long-axis systolic function was compromised, as exemplified by the averaged septal and lateral systolic annular velocities measured with pulse-wave tissue Doppler (s′ septal and s′ lateral, respectively), while in the younger (1.III.2, Fig. 1) and older brother (1.III.1, Fig. 1) it was preserved. In the case of right ventricular systolic function, the longitudinal tricuspid annular-plane systolic excursion (TAPSE) was only 7 mm, suggesting significant impairment of longitudinal right ventricular shortening. However, this was slightly better in the symptomatic brother (10 mm) and relatively good in the asymptomatic brother (17 mm).

Severe diastolic dysfunction with raised filling pressures was present in the proband, as shown by the restrictive transmitral filling pattern (transmitral E-wave deceleration time 96 ms) and E/e′ lateral > 10, in addition to the atrial dilatation. In individual 1.III.2, the left ventricular diastolic parameters were measured with the patient in atrial fibrillation and were impaired. In the older, asymptomatic brother (1.III.1, Fig. 1), the left ventricular diastolic function was significantly impaired with early diastolic lateral annular long-axis velocities of 7 cm/s. Filling pressures were elevated as evidenced by a restrictive transmitral filling pattern (transmitral pulse-wave E/A ratio of 2.7 and E-wave deceleration time of 111 ms) supported by an E/e′ lateral of almost 10.

The distribution of hypertrophy in/between the family members varied. The proband showed a focal area of significant hypertrophy involving the left ventricular apex and mid- to apical segments of the right ventricular free wall [maximal wall thickness (MWT) 14 mm]. In the younger brother (1.III.2, Fig. 1) the pattern of hypertrophy involved the right ventricle exclusively. The mid- to basal right ventricular free wall demonstrated significant focal hypertrophy of up to 10 mm, causing the bulging right ventricular free wall to almost ‘kiss’ the interventricular septum in systole – associated with a small intra-cavitary right ventricular gradient and prominent systolic flow turbulence on colour Doppler. The elder brother of the proband (1.III.1, Fig. 1) showed a focal area of hypertrophy with a MWT of 13 mm at the mid-septal region associated with mid-left ventricular cavity obliteration.

In pedigree 2, the proband (2.II.3, Fig. 3) was the only affected person from the family and showed systolic impairment with poor EF of 31%. In line with this, systolic left ventricular longaxis function and right ventricular long-axis systolic function, as assessed by a TAPSE of 6 mm, was severely impaired. Tissue Doppler indices demonstrated an individual with severe left ventricular diastolic early relaxation impairment and high filling pressures, reflected by a restrictive transmitral filling pattern (E/A; E-wave deceleration time and E/e′ lateral measuring > 13) (Table 2). Significantly more hypertrophy was observed in the MWT (21 mm) and mid-right ventricular free wall (9 mm). Furthermore, the right heart flow was sluggish as highlighted by marked spontaneous echo contrast present in both the right atrium and right ventricle.

## Molecular analysis

Molecular analysis of the cohort identified 14 genetic variants, of which six were exonic variants and eight were intronic variants (Table 3). In total, four novel variants were identified, of which one was an exonic variant (p.Leu144His) and three were intronic variants (c. –47C>T, c.109–17C>A, c.*35C>T). The remaining variants have previously been documented, and all exonic variants that resulted in an amino acid change (p.Pro82Ser,^9^,^10^ p.Arg162Gln^9^,^11^,^12^ p.Arg170Gln^11^) were tested for by restriction enzyme analysis in available family members.

**Table 3:** List of genetic variants identified in *TNNI3*

*Fragment*	*Sequence v ariants*	*Amino acid effect*	*Documented*
Exon 1	c.-148A>G	None	rs73935313
	c.-47C>T	None	Novel
	c.-35C>A	None	rs3729707
Exon 2	c.25-8T>A	None	rs3729836
	c.108+21G>A	None	rs3729837
Exon 4	c.109-17C>A	None	Novel
	c.150+13G>A	None	rs73617692
Exon 5	c.198G>A	p.Glu66	rs3729710
	c.204G>T	p.Arg68	rs3729711
	c.244C>T	p.Pro82Ser	rs77615401
Exon 7	c.432TG>AT	p.Leu144His	*Novel
	c.485G>A	p.Arg162Gln	Previously described^12^,^14^,^15^
	c.509G>A	p.Arg170Gln	Previously described^16^
Exon 8	c.*35C>T	None	Novel

Arg: arginine; Glu: glutamine; His: histadine; Leu: leucine; *TNNI3*: cardiac troponin I; Pro: proline. *Novel mutation caused by two adjacent sequence changes; c.432T>A (synonymous), which has been reported before,^5^ and a novel c.433G>T (non-synonymous) variant.

The mutation found in pedigree 1 (p.Leu144His) is novel, and together with the exonic variant (p.Arg170Gln) found in pedigree 2, have not been observed in the South African population before; these will be discussed in more detail. The remaining two identified exonic variants, namely p.Arg162Gln and p.Pro82Ser, were observed in two patients diagnosed with HCM. One patient presented with symptoms of atrial fibrillation (male, 61 years) and the other, an unexpected death, was diagnosed post mortem (male, 41 years, ventricular hypertrophy). Both mutations had previously been associated with HCM. Other family members were either unaffected by HCM or not available for further testing.

## Identification of a novel cardiac troponin I gene mutation

A *BccI* restriction enzyme digest was performed to confirm the presence of the p.Leu144His mutation in the three siblings in pedigree 1. Furthermore, this mutation was absent in a healthy, ethnically matched control group (*n* = 100). Genotypes of pedigree 1 for the p.Leu144His mutation were further confirmed by bi-directional semi-automated DNA sequencing (Fig. 1).

## Identification of previously documented *de novo* missense mutation

The mutation p.Arg170Gln (c.509G>A) has previously been associated with HCM in children younger than 13 years.^13^ A *PstI* restriction enzyme digestion confirmed that this mutation was not present in the other available family members of pedigree 2, including the parents of individual 2.II.3. Haplotype analysis of pedigree 2 derived from four microsatellite markers in the *TNNI3*-located chromosomal region shown in Fig. 3 indicated that the parents (2.I.1 and 2.I.2) are definitely the biological father and mother of all three of their offspring (2.II.1, 2.II.2 and 2.II.3). The p.Arg170Gln (c.509G>A) mutation is considered to have occurred *de novo* in the affected patient (2.II.3), since this mutation was absent in all other family members available for testing, and no family members presented with symptoms.

## Discussion

In this article we describe the screening of a panel of HCM-affected probands for *TNNI3*, a gene that has been associated not only with HCM but also with dilated cardiomyopathy and RCM.^4^ The screening was performed because two unrelated individuals, referred with HCM, had features resembling that of RCM.

Of the sequence variants identified, four caused amino acid changes, of which two were present in our probands with RCM; one novel (p.Leu144His) and one a previously described *de novo* mutation (p.Arg170Gln). The p.Leu144His mutation was present in the first actin-binding domain and overlapped with the ATPase inhibitory region, and p.Arg170Gln was located in the second actin-binding domain.^14^ The other two, namely p.Arg162Gln and p.Pro82Ser, were observed in two patients diagnosed with HCM. One patient presented with symptoms of atrial fibrillation (male, 61 years) and the other was an unexplained death diagnosed post mortem (male, 41 years). Both mutations have previously been associated with HCM. Other family members were either unaffected by HCM or not available for further testing.

RCM segregated in pedigree 1 for two generations, while in pedigree 2 an isolated case presented with RCM with no evidence of clustering within his immediate family. Two individuals also fulfilled the diagnostic criteria for HCM by way of focal ‘hypertrophy’ for HCM. The p.Leu144His mutation was also present in two brothers (1.III.1 and 1.III.2, Fig. 1) and most likely in the father of the proband who died from the same disease. The p.Arg170Gln mutation was identified in a single individual (2.II.3, Fig. 3). We have subsequently shown that this mutation arose *de novo* on the background of a haplotype inherited from his father. In a previous study investigating the genetic aetiology of HCM in pre-adolescent children between 1989 and 2007, the p.Arg170Gln mutation was the only mutation identified in *TNNI3*.^13^

The proband and her father in pedigree 1 were severely affected but only became symptomatic in their mid-twenties, and had had children before then. The other two siblings of the proband voluntarily had no children. In most cases of sarcomere-associated RCM, individuals with mutations in *TNNI3* in particular, present with likely de novo mutations, as affected individuals tend to die young.^3^,^4^ This pattern was observed in 2.II.3 (Fig. 3), who died at age 16 years, and had a *de novo* mutation on an allele inherited from his father.

The marked bi-atrial dilatation in the presence of normal-to-small left and right ventricular dimensions and generally preserved left ventricular systolic function (as measured by left ventricular ejection fraction in three of four cases) argues strongly for a restrictive process involving both ventricles in these cases. Left ventricular diastolic function measurements were universally abnormal, as expected, and this process associated with high filling pressures most likely explains the bi-atrial dilatation (inferior vena cava data for high right ventricular filling pressures – see Table 2). These ‘restrictive hearts’ show two additional morphological features of interest, namely focal hypertrophy, which often does not reach the cut-off values for the diagnosis of HCM by current criteria (15 mm), and prominent involvement of the right ventricle in terms of hypertrophy (three of four patients). The prominence of right ventricular involvement is not only echocardiographic, but translates into clinical involvement in terms of prominent right heart failure.

The index cases from both pedigrees demonstrated evidence of advanced long-axis systolic impairment of both ventricles and were worst affected from the viewpoint of symptomatic congestive heart failure. The elder brother of the proband from pedigree 1 (1.III.1, Fig. 1), with sparing of long-axis systolic function of both ventricles, seemed least affected clinically and in fact was relatively asymptomatic. The brother from pedigree 1 was intermediate, with relatively preserved left ventricular longaxis function but significantly impaired right ventricular longaxis function. The right ventricular long-axis function in all these cases, as assessed by TAPSE, appeared to predict the presence of right heart failure, or vice versa. Furthermore, interestingly, both these features tracked the presence of right ventricular hypertrophy in all three individuals exhibiting this.

Empirical data on outcomes related to right ventricular structure is generally lacking because of the unique geometry of the right ventricle.^15^ TAPSE estimates longitudinal function, but is not comprehensive.^15^ However, a recent magnetic resonance imaging-based study showed a higher risk in persons free of cardiovascular disease at baseline, but with an increased right ventricular mass.^16^

The novel mutation p.Leu144His involves the same amino acid residue as p.Leu144Gln, although it results in a different amino acid change. The latter is one of several RCM-causing mutations studied at laboratory level (p.Arg145Trp, p.Ala171Thr, p.Lys178Glu and p.Arg192His). The amino acid involving these mutations is located within the actin-binding domain (residue 130–148, 173–181) and the troponin C-binding domain (residue 113–164) of cardiac troponin I.^17^

In principle, this mutation could affect the normal function of cardiac troponin I since it is located in the sequence (residues 137–148) required for inhibition of human cardiac troponin I actomysin ATPase activity^18^ and has been shown to cause excessive inhibition in assays. However, all of the mutations tested, including p.Ala171Thr, which is in the second actin-binding domain and is next to the *de novo* mutation, p.Arg170Gln, also have similar effects on basal actin-myosin–ATPase activity. One suggestion is that the second binding domain increases the cardiac troponin I concentration on actin.^14^

All of these mutations exhibit an increased Ca^++^ sensitivity of the myofilament.^19^,^20^ Although it is not clear how this affects cross-bridge cycling and sarcomere energetics, some experiments suggest that these mutations are thought to weaken the ability of the cardiac troponin complex to fully inhibit the cross-bridge attachment during the relaxation phase of muscle contraction,^21^–^23^ consequently reducing the rate of relaxation. Higher energy use observed in transgenic models of HCM at a whole-cell or whole-animal level may relate to a shift in Ca^++^ homeostasis (or other changes) with higher energy costs to return the cells to their pre-contractile basal state.

## Conclusion

In the current investigation, we have demonstrated the usefulness of HRM analysis for the identification of HCM-causing mutations. The mutations identified here will now be used for pre-symptomatic genetic screening of additional family members to identify individuals at risk. Furthermore, new patients with similar clinical features could also initially be screened for these mutations.

Research studies often do not recount diagnostic disparities, which could be a result of high incidence of disease, diagnostic sophistication and personal interests. The ‘wrong’ diagnoses of cor pulmonale, CP and TB pleuritis highlight the influence of the high prevalence of TB in South Africa in making a diagnosis. Furthermore, this study provides insight into diagnostic disparities and processes that led to inappropriate diagnosis in two individuals.
